# Integrated analysis identified the role of three family members of ARHGAP in pancreatic adenocarcinoma

**DOI:** 10.1038/s41598-024-62577-z

**Published:** 2024-05-23

**Authors:** Haoran Fei, Xiao Shi, Dan Sun, Haishen Yang, Dali Wang, Kai Li, Xinxin Si, Wei Hu

**Affiliations:** 1 Department of Hepatobiliary Surgery, The First People’s Hospital of Lianyungang, The First Affiliated Hospital of Kangda College of Nanjing Medical University, Lianyungang, 222000 Jiangsu China; 2 Jinzhou Medical University, Jinzhou, 121001 Liaoning China; 3 Jiangsu Key Laboratory of Marine Pharmaceutical Compound Screening, College of Pharmacy, Jiangsu Ocean University, Lianyungang, 222005 Jiangsu China

**Keywords:** Cancer, Computational biology and bioinformatics

## Abstract

The Rho GTPase activating protein family (ARHGAPs) is expressed in pancreatic adenocarcinoma (PAAD) but its function is unclear. The aim of this study was to explore the role and potential clinical value of ARHGAPs in PAAD. Using TCGA and GEO databases to analyze expression of ARHGAPs in PAAD and normal tissues. Survival curve was drawn by Kaplan–Meier. ARHGAPs were integrated analyzed by GEPIA2, TIMER, UCLCAN, cBioPortal and R language. Protein level and prognostic value were evaluated via IHC staining or survival analysis. We totally identify 18 differentially expressed (DE) ARHGAPs in PAAD. Among the 18 DE genes, 8 were positively correlated with tumor grade; abnorrmal expression of 5 was positively correlated with copy number variation; expression of 4 was positively correlated with promoter hypomethylation. Multivariate Cox regression identified ARHGAP5, ARHGAP11A, and ARHGAP12 as independent prognostic factors of PAAD. The function of ARHGAPs was mainly related to GTPase activity and signaling, axon guidance, proteoglycans in cancer and focal adhesion. Expression of 7 ARHGAPs was strongly correlated with immune infiltration. Immunohistochemistry showed increased protein levels of ARHGAP5, ARHGAP11A, and ARHGAP12 in PAAD tissues. Survival analysis confirmed a negative correlation between ARHGAP5, ARHGAP11A, and ARHGAP12 expression and patient prognosis. Multivariate Cox regression proved ARHGAP5, ARHGAP11A, and ARHGAP12 could serve as independent prognostic indicators for PAAD. Finally, this study verified ARHGAP5, ARHGAP11A, and ARHGAP12 as independent prognostic factors in PAAD, suggesting their significance for the diagnosis and treatment of PAAD.

## Introduction

Pancreatic adenocarcinoma (PAAD), one of the common malignant tumors of the digestive tract, is characterized by late diagnosis and poor prognosis^1^. Despite improvements in tumor treatment technology and the increasingly better prognosis of cancer patients, the treatment of PAAD still faces great challenges, especially in patients with advanced disease at diagnosis. No effective prognostic biomarkers are available that can be used for PAAD diagnosis^2^; therefore, searching for good predictive indicators and exploring their roles in PAAD remain significant research focuses.

The Rho GTPase activating protein family members (ARHGAPs) may be one group of potential PAAD indicators. ARHGAPs i one of the major regulators of Rho GTPase, an important intracellular signaling molecule that perceives a variety of stimulation signals and participates in cytoskeleton reorganization, the cell cycle, cell adhesion and migration, substance transport, and regulation of several physiological processes^3^. ARHGAPs are negative mediators of Rho GTPases and exert their functions by catalyzing the conversion of the active GTP-bound state of Rho GTPases to the inactive GDP-bound state^4^. Recently, abnormal expression of ARHGAPs was reported in a variety of tumors and was closely related to the occurrence and development of tumors. One ARHGAP, DLC1 (ARHGAP7), suppresses cell proliferation, anchorage-independent growth, and in vivo tumorigenicity of hepatocellular carcinoma by negatively regulating the activity of Rho proteins^5^.Conversely, by gaining fusion genes, ARHGAP6/ARHGAP26 fusions decrease cell apoptosis in gastric cancer^6^. Roles for several ARHGAP family members have been identified in several types of cancer^7–13^, but the precise function of ARHGAP family members in PAAD, and especially in the immunological microenvironment of PAAD, has not been elucidated.

Current advancements in computer technology and the availability of public databases have allowed the study of cancer genomics with sequencing data and clinical information contained in these databases. Two of the most used bioinformatics databases are the cancer genome atlas (TCGA) and the gene expression omnibus (GEO)^14,15^. These databases provide the ability to conduct a wide range of genomic analyses, such as expression analysis, survival analysis, and immunological microenvironment analysis^16^. The present study was an investigation of the expression, prognostic significance, and immunological connection of the entire range of ARHGAP family members in PAAD. Our findings could lead to new approaches to PAAD treatment.

## Results

### Differential expression of ARHGAP family members in PAAD patients

**Figure 1:**
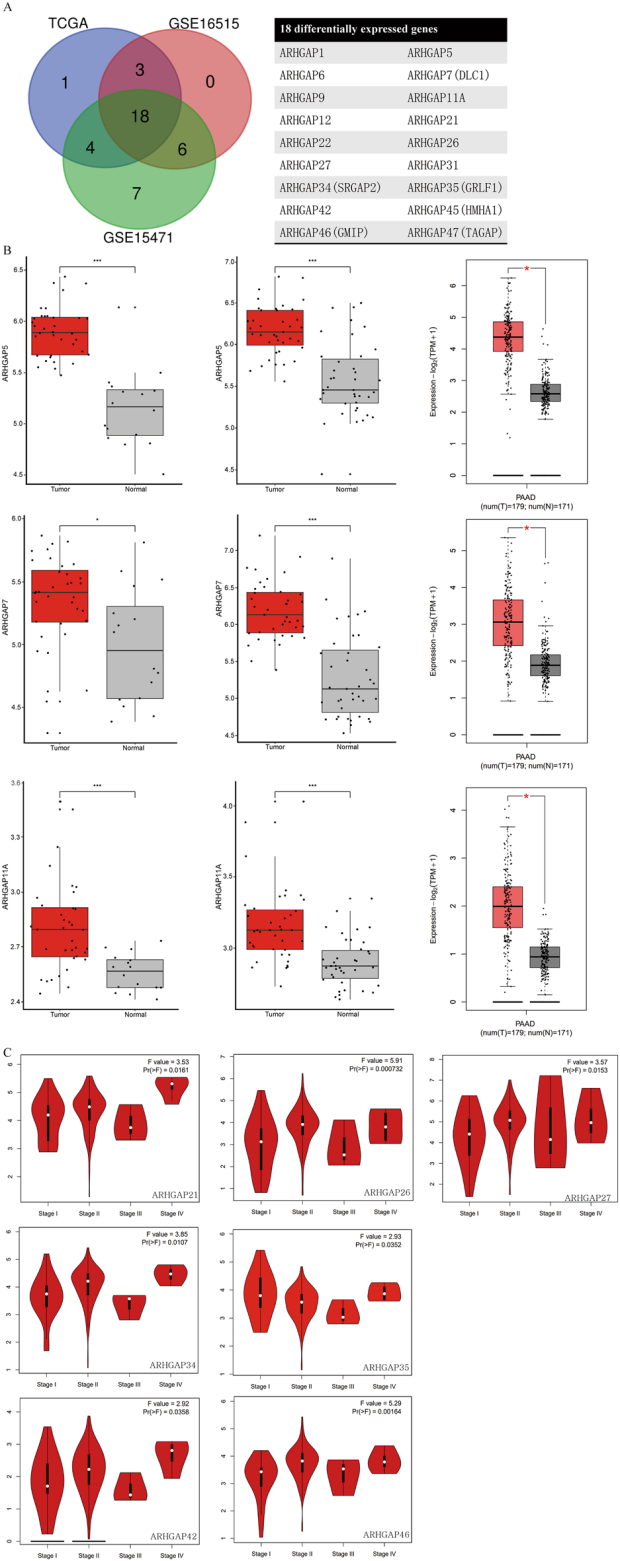
Differential expression of ARHGAP family members in PAAD patients. (**A**) Left panel shows the Venn diagram of the differentially expressed genes in the three datasets. Right panel lists the 18 upregulated genes in all three datasets. (**B**) The expression level of representative ARHGAPs in PAAD tissues from GSE16515, GSE15471, and GEPIA2. (**C**) Correlation between tumor stages and expression of ARHGAPs.

Based on the data from the GSE16515, GSE15471, and TCGA, we first explored the expression of 49 ARHGAP members in PAAD tumor and normal tissues. The GSE16515 data revealed increased expression of 27 ARHGAPs, the GSE15471 data revealed increased expression of 35 ARHGAPs, and the TCGA data revealed increased expression of 26 ARHGAPs in tumor tissues. A total of 18 ARHGAPs were upregulated in all three datasets (Fig. 1A): ARHGAP1, ARHGAP5, ARHGAP6, ARHGAP7 (DLC1), ARHGAP9, ARHGAP11A, ARHGAP12, ARHGAP21, ARHGAP22, ARHGAP26, ARHGAP27, ARHGAP31, ARHGAP34 (SRGAP2), ARHGAP35 (GRLF1), ARHGAP42, ARHGAP45 (HMHA1), ARHGAP46 (GMIP), and ARHGAP47 (TAGAP). Figure 1B shows the differential expression in the three datasets for 3 representative genes. The correlations between the expression of the 18 differentially expressed (DE) ARHGAPs and the pathological stage were analyzed using GEPIA2. A significant association was detected between ARHGAP21, ARHGAP26, ARHGAP27, ARHGAP 34, ARHGAP 35, ARHGAP42, and ARHGAP46 and the tumor stages (Fig. 1C).

### Genetic variations and promoter methylation of ARHGAPs in PAAD

**Figure 2:**
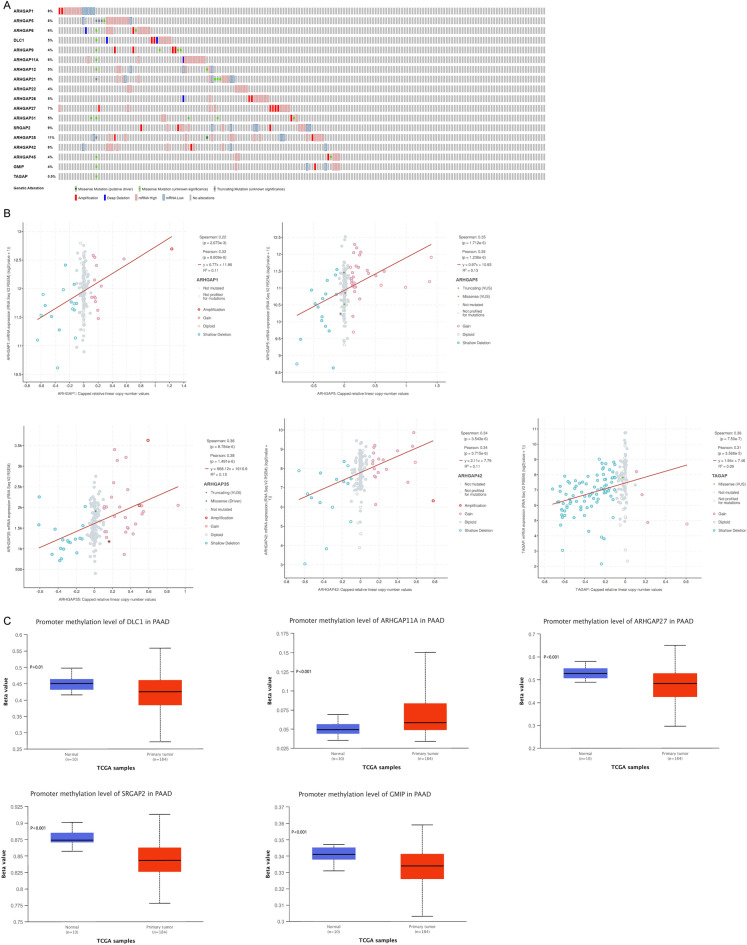
Genetic variations and promoter methylation of ARHGAPs in PAAD. (**A**) Summary of genetic alterations of different expressed ARHGAPs in PAAD. (**B**) Relationship between copy number alternations (CNA) in ARHGAPs and expression of mRNA. (**C**) The methylation levels of ARHGAP7 (DLC1), ARHGAP11A, ARHGAP27, ARHGAP34 (SRGAP2), and ARHGAP (GMIP) are downregulated in tumor tissues.

We explored genetic variations of ARHGAPs using cBioPortal. The ARHGAP mutation profiles were obtained from Pancreatic Adenocarcinoma (TCGA, Firehose Legacy) with 186 patients. A high ARHGAP genetic variation was observed in PAAD patients (Fig. 2A). Among all ARHGAPs, ARHGAP35 (GRLF1) is regarded as the top-ranking gene in terms of genetic alteration in PAAD patients (11%). The correlation between ARHGAP copy number alterations (CNA) and expression of mRNA, as shown in Fig. 2B, indicated a positive correlation between the copy numbers of ARHGAP1, ARHGAP5, ARHGAP35 (GRLF1), ARHGAP42, and ARHGAP47 (TAGAP) and the mRNA expression in PAAD. Promoter methylation analysis also revealed a relationship between high expression of ARHGAP7 (DLC1), ARHGAP11A, ARHGAP27, ARHGAP34 (SRGAP2), and ARHGAP46 (GMIP) and decreased methylation level (Fig. 2C).

### Prognostic value of the expression of ARHGAPs in PAAD patients

**Figure 3:**
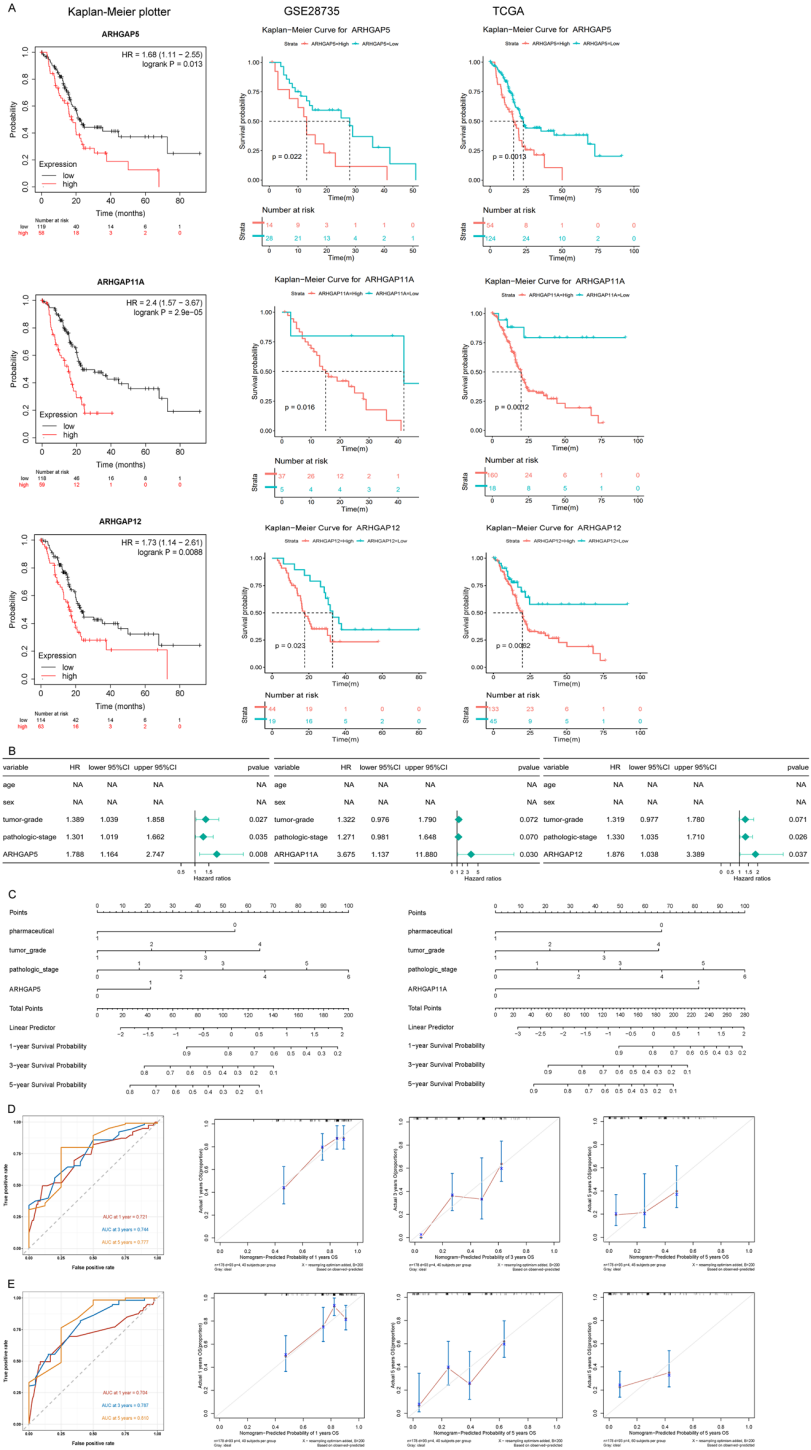
Prognostic value of the expression of ARHGAPs in PAAD patients. (**A**) High expression of ARHGAP5, ARHGAP11A, and ARHGAP12 is correlated with short OS in all three datasets. (**B**) Multivariate Cox regression analyses were used to estimate the risk factors of ARHGAP5, ARHGAP11A, and ARHGAP12 for PAAD. (**C**–**E**) The ROC area under the curve and the calibration curve of ARHGAP5 and ARHGAP11A.

We next evaluated the prognostic value of the 18 DE ARHGAPs and the progression of PAAD, the correlations between different ARHGAPs, and clinical outcomes. The expression of ARHGAP5, ARHGAP11A, and ARHGAP12 was negatively correlated with overall survival (OS) in all three datasets (Fig. 3A).

We verified the prognostic value by performing univariate and multivariate Cox regression analyses. The multivariate Cox regression analyses showed that ARHGAP5, ARHGAP11A, and ARHGAP12 were independent predictors of survival in PAAD (Fig. 3B). Nomogram construction based on independent prognostic factors to predict the individual survival probability. (Fig. 3C) revealed that the 1-, 3-, and 5-year survival rates for each patient would be predicted by the total points in the nomogram according to the indicators. We assessed the sensitivity and specificity of this nomogram using time-dependent receiver operating characteristic (ROC) analysis. The ROC area under the curve (AUC) of ARHGAP5 was 0.721 for 1-year survival, 0.744 for 3-year survival, and 0.777 for 5-year survival, representing an efficient predictive efficacy (Fig. 3D). Application of a calibration curve to evaluate the nomogram accuracy indicated that the nomogram could effectively predict the prognosis of PAAD patients (Fig. 3D). The ROC area under the curve and the calibration curve of ARHGAP11A are shown in the picture (Fig. 3E).

### Gene ontology enrichment and gene set enrichment analyses

**Figure 4:**
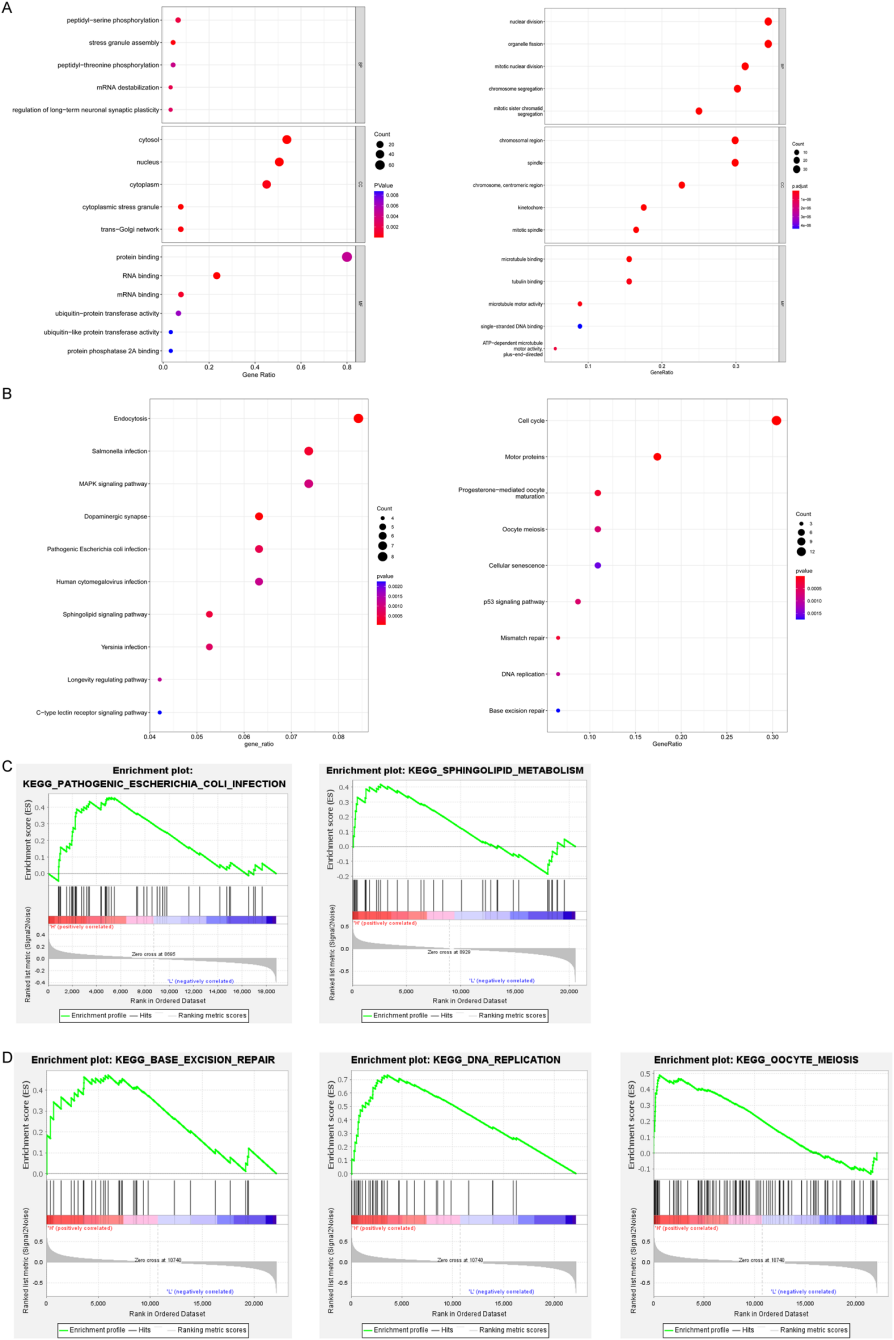
Gene ontology enrichment and gene set enrichment analyses. (**A**) All GO enrichment analysis results for ARHGAP5 (left panel) or ARHGAP11A (right panel) and the interaction proteins. *MF* molecular function; *CC* cellular component; *BP* biological process. (**B**) All KEGG pathway enrichment results for the ARHGAP5 (left panel) or ARHGAP11A (right panel) and the interaction proteins. (**C**) GSEA showed that pathogenic *Escherichia coli* infection and sphingolipid metabolism were significantly enriched in the high ARHGAP5 expression group. (**D**) GSEA showed that base excision repair, DNA replication, and oocyte meiosis pathway were significantly enriched in the high ARHGAP11A expression group.

We used the “Similar Genes Detection” module of GEPIA2 to obtain the top 100 ARHGAP5 or ARHGAP11A correlated genes based on the datasets of all TCGA tumor and normal tissues (supplementary materials Table S1). The 100 genes and ARHGAP5 or ARHGAP11A were then subjected to GO and KEGG analysis. The GO results revealed that the function of ARHGAP5 was associated with protein binding, cytosol, and peptidyl-serine phosphorylation functions, whereas the function of ARHGAP11A was associated with nuclear division, chromosomal region, and microtubule binding (Fig. 4A). The KEGG analysis showed strong correlations of ARHGAP5 with endocytosis, salmonella infection, the MAPK signaling pathway, and the sphingolipid signaling pathway, whereas ARHGAP11A was correlated with the cell cycle, cellular senescence, and the p53 signaling pathway (Fig. 4B). GSEA further confirmed significant enrichment in pathogenic *Escherichia coli* infection and sphingolipid metabolism in the ARHGAP5 high-expression group (Fig. 4C). Base excision repair, DNA replication, and oocyte meiosis were significantly enriched in the ARHGAP11A high-expression group (Fig. 4D).

### Analysis of the immune microenvironment

**Figure 5:**
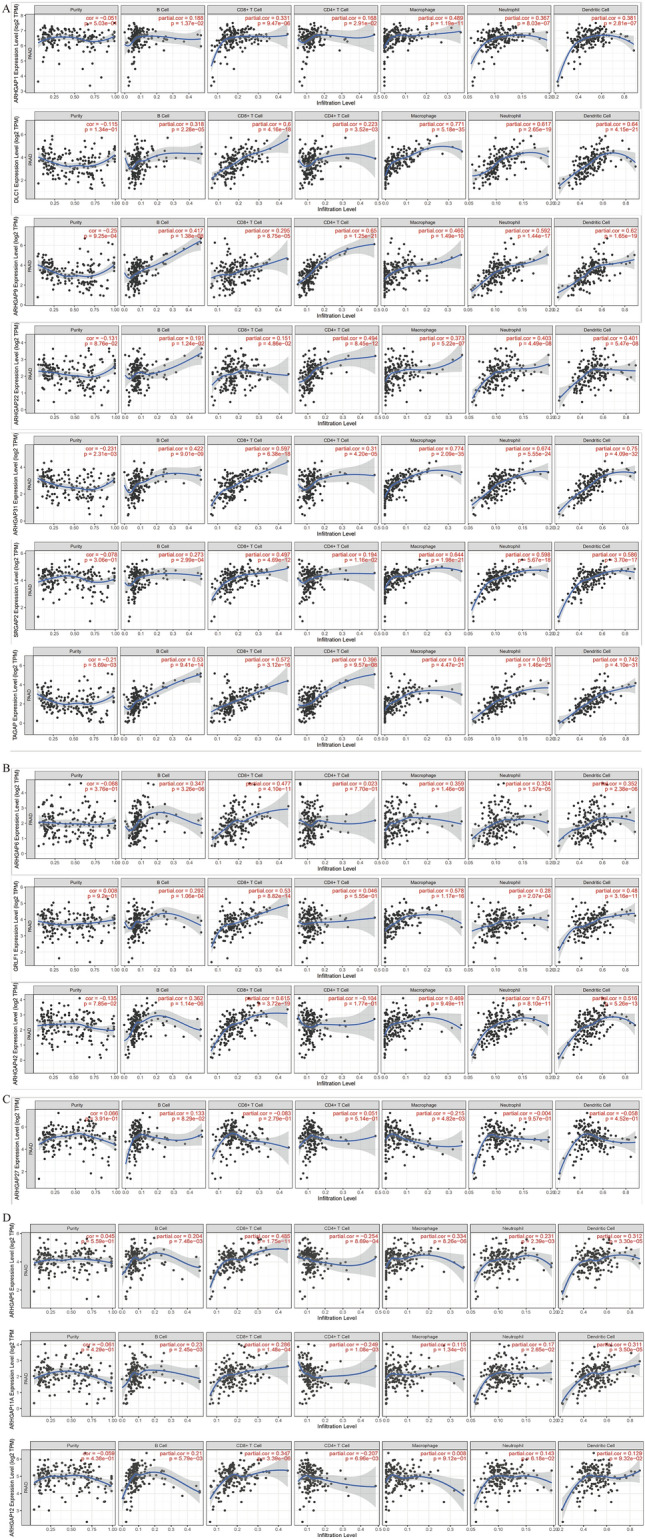
Analysis of the immune microenvironment. (**A**–**D**) The expression correlation between distinct ARHGAPs and immune cells, as analyzed using TIMER. The *p* value is shown in the figures. Partial.cor: purity-corrected partial Spearman’s rho value.

The immune cell level is associated with the proliferation and progression of cancer cells. We used the TIMER database to explore the correlation between the 18 DE ARHGAPs and immune cell infiltration. ARHGAP1, ARHGAP7 (DCL1), ARHGAP9, ARHGAP22, ARHGAP31, ARHGAP34 (SRGAP2), and ARHGAP47 (TAGAP) were positively correlated with infiltration of B cells, CD8 + T cells, CD4 + T cells, macrophages, neutrophils, and dendritic cells in PAAD (Fig. 5A). Expression of ARHGAP6, ARHGAP35 (GRLF1), and ARHGAP42 was positively associated with the infiltration of B cells, CD8 + T cells, macrophages, neutrophils, and dendritic cells in PAAD (Fig. 5B). ARHGAP27 expression was only negatively correlated with the infiltration of macrophage cells (Fig. 5C). ARHGAP5 expression was positively associated with B cells, CD8 + T cells, macrophages, neutrophils, and dendritic cells, but negatively correlated with CD4 + T cells. ARHGAP11A expression was positively associated with B cells, CD8 + T cells, neutrophils, and dendritic cells, but negatively correlated with CD4 + T cells. ARHGAP12 expression was positively associated with B cells and CD8 + T cells, but negatively correlated with CD4 + T cells (Fig. 5D).

### Immunotherapy outcome prediction

**Figure 6:**
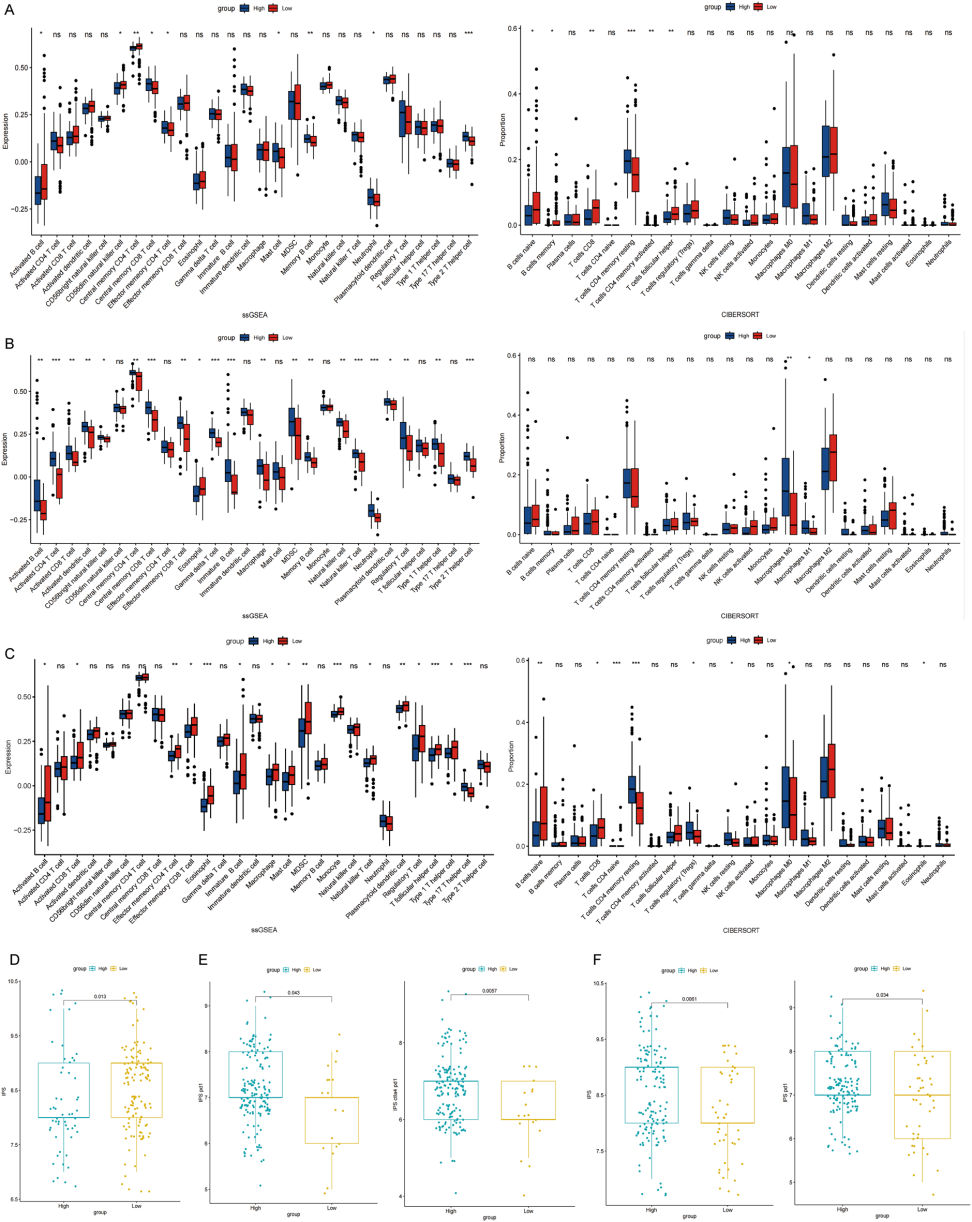
Immunotherapy outcome prediction. (**A**) The differences in immune cell infiltration levels between the high and low ARHGAP5 expression groups from ssGSEA or CIBERSORT. (**B**) The differences in immune cell infiltration levels between the high and low ARHGAP11A expression groups from ssGSEA or CIBERSORT. (**C**) The differences in immune cell infiltration levels between the high and low ARHGAP12 expression groups from ssGSEA or CIBERSORT. (**D**) The IPS based on different ARHGAP5 expression. *IPS* immunophenoscore. (**E**) The IPS of PD1 or CLTA4 PD1 based on different ARHGAP11A expression. (**F**) The IPS or IPS of PD1 based on different ARHGAP12 expression.

We further confirmed the role of the ARHGAP5, ARHGAP11A, and ARHGAP12 in immunotherapy by calculating the degree of infiltration of 28 immune cell types in high or low ARHGAP5, ARHGAP11A, or ARHGAP12 in PAAD samples. A close relationship was evident between ARHGAP5 and memory B cells, CD4 T cells, and CD8 T cells (Fig. 6A). A close relationship was also apparent between ARHGAP11A and macrophages (Fig. 6B). A close relationship was detected between ARHGAP12 and macrophages, eosinophils, and regulatory T cells (Fig. 6C). Immunotherapy outcome predictions showed that the low ARHGAP5 group had a higher immunophenoscore (IPS) (Fig. 6D). The expression of ARHGAP11A was positively correlated with PD1 or CTLA4 PD1 IPS (Fig. 6E). The expression of ARHGAP12 was positively correlated with IPS or PD1 IPS (Fig. 6F).

### Predicted target drug therapy outcome

**Figure 7:**
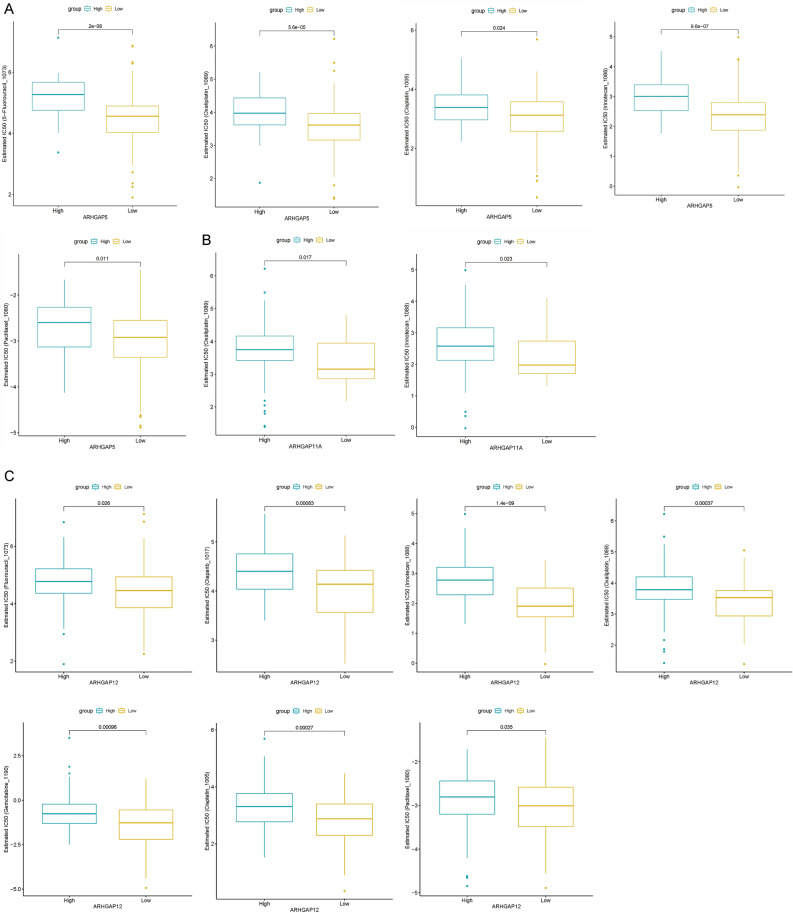
Predicted target drug therapy outcome. (**A**–**C**) GDSC predicts the IC_50_ of different drugs between PAAD patients with low or high expression levels of ARHGAP5, ARHGAP11A, and ARHGAP12.

Investigation of the correlation between ARHGAP5, ARHGAP11A, or ARHGAP12 and targeted drug sensitivity revealed an increased half maximum inhibitory concentration (IC_50_) for 5-fluorouracil, oxaliplatin, cisplatin, irinotecan, and paclitaxel in the high ARHGAP5 expression group (Fig. 7A). The IC_50_ values of oxaliplatin and irinotecan were also increased in the high ARHGAP11A expression group (Fig. 7B). The IC_50_ for fluorouracil, olaparib, oxaliplatin, irinotecan, gemcitabine, cisplatin, and paclitaxel were increased in the high ARHGAP11A expression group (Fig. 7C).

### Validation of the protein level and prognostic value of ARHGAP5, ARHGAP11A, and ARHGAP12 in PAAD tissues

**Figure 8:**
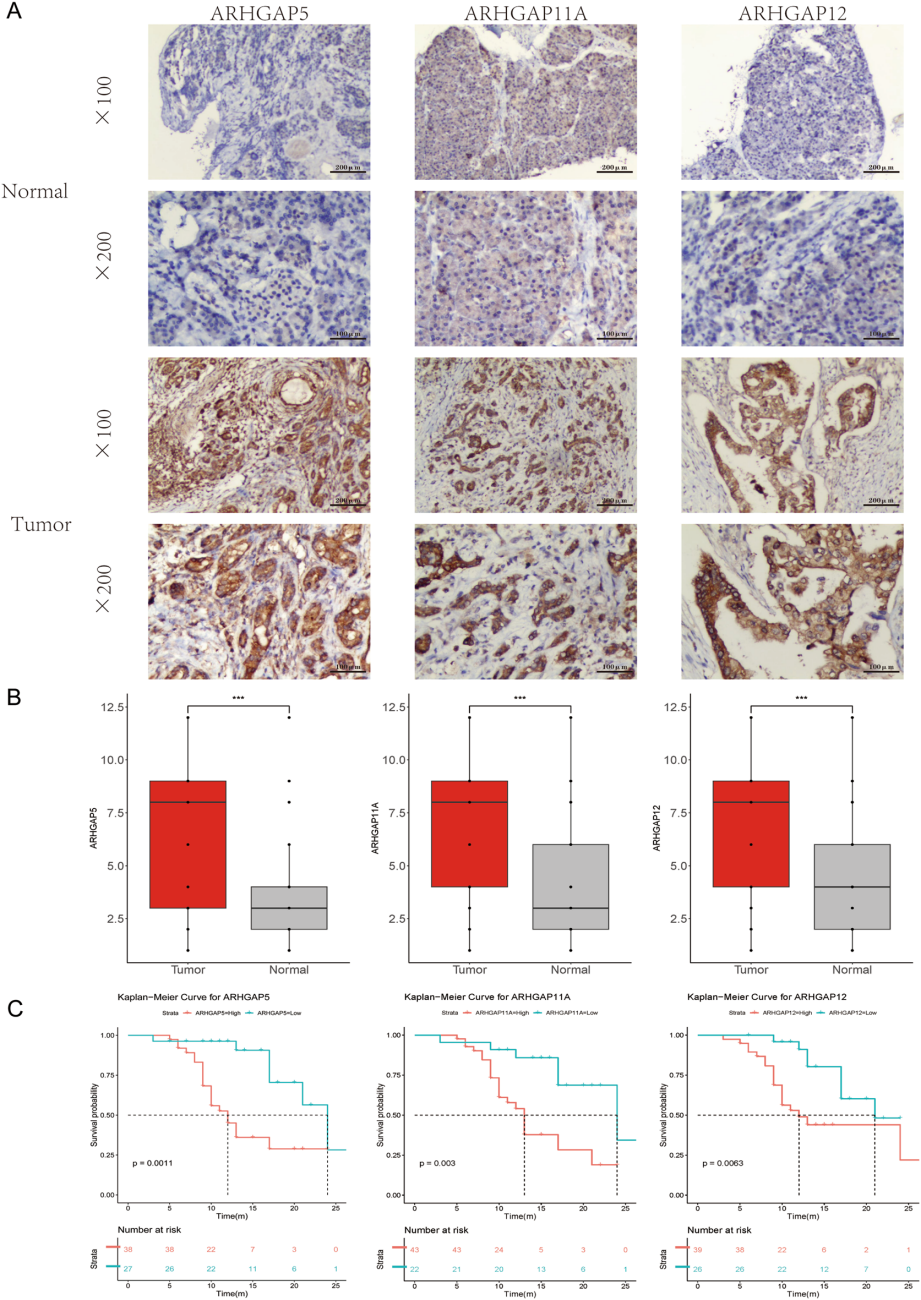
Validation of the protein level and prognostic role of ARHGAP5, ARHGAP11A, and ARHGAP12 in PAAD tissues. (**A**) Representative immunohistochemistry (IHC) images in PAAD and normal tissues. (**B**) Boxplot shows significant upregulation of ARHGAP5, ARHGAP11A, and ARHGAP12 compared to normal pancreas tissues. n (Tumor) = 65, n (Normal) = 65. (**C**) Expression of ARHGAP5, ARHGAP11A, and ARHGAP12 in PAAD pathology samples was negatively correlated with OS.

We chose the three potential predictors ARHGAP5, ARHGAP11A, and ARHGAP12 to validate expression and clinical prognosis in the PAAD and normal samples. Detailed clinical sample information, including age, gender, and pathological staging, has been provided in the supplementary materials Table S2. Immunohistochemistry results revealed higher ARHGAP5, ARHGAP11A, and ARHGAP12 expression in PAAD tissues than in normal tissues (Fig. 8A,B). The correlation of the clinical pathological characteristics and protein expression levels are summarized in Table 1. The protein expression level of ARHGAP5 was related to tumor size (*p* = 0.045), lymph node metastasis (*p* < 0.001) and tumor stage (*p* < 0.001). The expression level of ARHGAP11A was correlated with tumor size (*p* = 0.001), and tumor stage (*p* = 0.029). The expression level of ARHGAP12 was correlated with tumor size (*p* = 0.025). Overall survival analysis, as shown in Fig. 8C, revealed shorter overall survival in patients with high ARHGAP5, ARHGAP11A, and ARHGAP12 protein expression than with low expression of these proteins. Tumor stage, differentiation degree, and ARHGAP5 expression were significantly correlated with survival. The multivariate Cox regression analyses showed that differentiation degree and expression of ARHGAP5, ARHGAP11A, and ARHGAP12 were independent predictors of survival in PAAD (Tables 2, 3 and 4). Taken together, these findings demonstrated a potential prognostic significance for ARHGAP5, ARHGAP11A, and ARHGAP12 expression in clinical patients.

**Table 1:** Univariate analysis of the protein level and clinicopathological characteristics.

Clinicopathological features	Case	ARHGAP5			ARHGAP11A			ARHGAP12		
		Low (n = 27)	High (n = 38)	P value	Low (n = 22)	High (n = 43)	P value	Low (n = 26)	High (n = 39)	P value
Age										
< 65	28	13	15	0.486	8	20	0.434	8	20	0.102
≥ 65	37	14	23		14	23		18	19	
Gender										
Female	26	10	16	0.681	11	15	0.239	12	14	0.408
Male	39	17	22		11	28		14	25	
Tumor size										
< 3	29	16	13	0.045	16	13	0.001	16	13	0.025
≥ 3	36	11	25		6	30		10	26	
Lymph node metastasis										
No	37	23	14	0.001	15	22	0.190	16	21	0.540
Yes	28	4	24		7	21		10	18	
Tumor stage										
I–IIA	32	22	10	< 0.001	15	17	0.029	16	16	0.105
IIB–IV	33	5	28		7	26		10	23	
Differentiation degree										
Low	11	2	9	0.338	1	10	0.082	2	9	0.177
Intermediate–high	54	24	30		21	33		24	30	
Perineural invasion										
No	33	15	18	0.515	13	20	0.337	14	19	0.685
Yes	32	12	20		9	23		12	20

**Table 2:** Univariate and multivariate Cox regression analyses of ARHGAP5.

Characteristics	Univariate analysis			Multivariate analysis		
	P	HR	95% CI	P	HR	95% CI
Age	0.567	0.8	0.373–1.717			
Gender	0.742	0.88	0.41–1.888			
Tumor size	0.069	2.122	0.944–4.77			
Lymph node metastasis	0.082	1.991	0.917–4.325			
Tumor stage	0.003	3.524	1.519–8.179	0.125	2.138	0.810–5.643
Differentiation degree	0.011	0.317	0.131–0.764	0.006	0.271	0.107–0.691
Perineural invasion	0.941	0.971	0.448–2.106			
ARHGAP5	0.003	4.014	1.60–10.074	0.030	3.256	1.121–9.461

**Table 3:** Univariate and multivariate Cox regression analyses of ARHGAP11A.

Characteristics	Univariate analysis			Multivariate analysis		
	P	HR	95% CI	P	HR	95% CI
Age	0.567	0.8	0.373–1.717			
Gender	0.742	0.88	0.41–1.888			
Tumor size	0.069	2.122	0.944–4.77			
Lymph node metastasis	0.082	1.991	0.917–4.325			
Tumor stage	0.003	3.524	1.519–8.179	0.01	3.149	1.310–7.569
Differentiation degree	0.011	0.317	0.131–0.764	0.035	0.37	0.147–0.934
Perineural invasion	0.941	0.971	0.448–2.106			
ARHGAP11A	0.007	3.64	1.435–9.23	0.047	2.682	1.014–7.096

**Table 4:** Univariate and multivariate Cox regression analyses of ARHGAP12.

Characteristics	Univariate analysis			Multivariate analysis		
	P	HR	95% CI	P	HR	95% CI
Age	0.567	0.8	0.373–1.717			
Gender	0.742	0.88	0.41–1.888			
Tumor size	0.069	2.122	0.944–4.77			
Lymph node metastasis	0.082	1.991	0.917–4.325			
Tumor stage	0.003	3.524	1.519–8.179	0.01	3.205	1.327–7.740
Differentiation degree	0.011	0.317	0.131–0.764	0.019	0.327	0.129–0.834
Perineural invasion	0.941	0.971	0.448–2.106			
ARHGAP12	0.011	3.112	1.302–7.437	0.026	2.934	1.136–7.574

## Discussion

We systematically investigated the expression, survival analysis, and function of the ARHGAP family proteins in pancreatic cancer, and we validated the three key genes in clinical tissues. We identified three novel prognostic factors (ARHGAP5, ARHGAP11A and ARHGAP12) for PAAD.

The Rho family small GTPases, such as RhoA, Rac1, and Cdc42 intracellular signaling molecules, are categorized into the Ras superfamily^17^. The activity of Rho GTPases is modulated by three types of protein (Rho-selective guanine nucleotide exchange factors (RhoGEFs), GTPase-activating proteins (RhoGAPs), and guanine nucleotide dissociation inhibitors (RhoGDIs)^18^. RhoGAPs are usually negative regulators of Rho GTPase activity; that is, they stimulate Rho proteins to assume an inactive GDP-bound state^19^. Although the traditional interpretation is that an inactivation of GTPase activity would suppress tumorigenesis, recent studies have raised doubts about this^20^.

ARHGAP5 (also named p190-B RhoGAP and belonging to the RhoGAP family) is a protein that negatively regulates the activity of RhoA^21^. ARHGAP5 can also promote cancer progression regardless of the activation state of RhoA. For example, high ARHGAP5 expression is associated with aggressive behavior of non-small-cell lung carcinoma, while the non-coding RNA derived from ARHGAP5 can inhibit breast cancer migration^22^ and the circular RNA produced by ARHGAP5 can inhibit cisplatin resistance in cervical squamous-cell carcinoma^7^. However, a function for ARHGAP5 in pancreatic cancer has not been previously reported.

Our use of bioinformatics tools in the present study identified ARHGAP5 as having important prognostic value in PAAD and validated this in clinical tissues. We also found a positive correlation between the expression levels of ARHGAP5 and its copy number variations, revealing a potential reason for the overexpression of ARHGAP5 in pancreatic cancer (Fig. 2B). More experimental studies are still needed to explore the mechanisms by which dysregulation of ARHGAP5 expression leads to the progression of PAAD.

ARHGAP11A is another member of the RhoGAP family, and its role in tumors is still controversial. ARHGAP11A induced cell cycle arrest and inhibited glioma cell growth by binding to p53 and increasing its activity^23^. Xiaoying Guan et al., found that ARHGAP11A enhanced the stability of actin microfilaments and tumor genesis via TPM1 in gastric cancers^24^. In the current study, we found that ARHGAP11A was overexpressed in PAAD tissues and negatively correlated with overall survival. The clinical tissue validation also confirmed the oncogene role of ARHGAP11A in PAAD.

ARHGAP12 has been found to negatively regulate Rac1 signaling^25^. In the brain, ARHGAP12 also plays an important role in synaptic structure and function in the developing hippocampus^26^. By contrast, little is known about the role of ARHGAP12 in cancer. In hepatocytes, ARHGAP12 inhibited cell invasion and adhesion to fibronectin in response to hepatocyte growth factor by inactivating Rac1^27^. In the present study, our informatics analysis and clinical tissue validation revealed that ARHGAP12 was an oncogene in PAAD.

Although the mechanism of action of ARHGAP5 and ARHGAP11A in pancreatic cancer is unclear, some oncogenic mechanisms have been identified in other tumors. In hepatocellular carcinoma, ARHGAP5 enhances cell spreading and migration by negatively modulating RhoA^28^. ARHGAP5 drives colorectal cancer metastasis through the negative regulation of RhoA activity^9^. ARHGAP11A facilitates the malignant advancement of gastric cancer by modulating actin filament stability via TPM1^24^. ARHGAP11A drive malignant progression through inactivating Rac1B in hepatocellular carcinoma^11^. ARHGAP11A enhanced the progression of breast cancer by facilitating cell cycle transition from the G1 to S phase^20^. These studies provide insights into the mechanisms of action of ARHGAP5 and ARHGAP11A in pancreatic cancer.

Although immunotherapy is largely ineffective in patients with PAAD, tumor immune microenvironment is prognostically instructive in PAAD. Some studies have already been conducted to try to find prognostic signature related to the specific immune microenvironment of PAAD^29–31^. Our findings reveal a significant correlation between the expression of ARHGAPs genes and the immune markers of tumor-associated macrophages (TAM), M1 and M2 macrophages, as well as subgroups of CD4 + T cell differentiation, including Th1, Th2, Tfh, Th17, and Treg cells (supplementary materials Table S4). Therefore, we also conducted immunotherapy outcome predictions or immune infiltrate-related analyses on the screened genes, aiming to identify effective predictive markers for immunotherapy in PAAD. Our results identified three potential predictive markers (ARHGAP5, ARHGAP11A, ARHGAP12) for the efficacy of immunotherapy in PAAD. ARHGAP11A was found to promote the proliferation and migration of renal cell carcinoma by inhibiting the tumor immune microenvironment^32^. Whether ARHGAP11A can promote the progression of pancreatic cancer by modulating the tumor immune microenvironment is a direction worth exploring in the future.

## Conclusions

In summary, we conducted a systematic and comprehensive analysis of the function of ARHGAP family members in PAAD. Three potential prognostic markers (ARHGAP5, ARHGAP11A, and ARHGAP12) for pancreatic cancer were identified. Although we have confirmed the function of three genes (ARHGAP5, ARHGAP11A, and ARHGAP12) at the histological level, further studies are still needed to explore the underlying molecular mechanisms.

## Materials and methods

### Data collection and analysis

The expression data and corresponding clinical information of PAAD patients were acquired from The Cancer Genome Atlas (TCGA) database and Gene Expression Omnibus (GEO) database (http://www.ncbi.nlm.nih.gov/geo/). The TCGA data were downloaded from the UCSC Xena database (https://xenabrowser.net/datapages/) and the GEO data included GSE28735, GSE15471, and GSE16515. All data were log2 transformed for subsequent analysis and then expression analyzed with the “limma” package in R version: 4.2.2. P-values < 0.05 were considered statistically significant. The information of the patients in TCGA and GEO were provided as supplementary materials (Table S3).

### Gene expression analysis

GEPIA2 (http://gepia2.cancer-pku.cn/#index), an upgraded version of GEPIA, is an interactional website application consisting of data from thousands of tumors and normal tissue samples and can be used to visualize clinicopathological characteristics. The tumor data came from the TCGA database. In this study, GEPIA 2 was used to compare tumor and normal tissues and for pathological staging and gene detection analysis. An independent *t*-test was used to calculate p-values, with p < 0.05 considered statistically significant; Pr (> F) < 0.05 was based on Student’s *t*-test.

### Survival analysis and Cox regression analysis

Kaplan–Meier (K-M) survival analysis was used to assess the differences between the high-expression group and the low-expression group, based on the best cutoff value expression of ARHGAP family genes through the “survminer” and “survival” packages in R. In this study, the prognostic values of the effect of ARHGAP family genes on the overall survival of patients with PAAD were also estimated using the Kaplan–Meier plotter (http://www.kmplot.com/). Patients were divided into a group with high expression and group with low expression according to the best cutoff. Univariate and multivariate Cox regression analyses were then conducted to explore the relationships between ARHGAP expression, clinical prognostic indicators, and survival time in TCGA-PAAD patients, generated via the “survival” and “forestplot” packages in R, with p-values < 0.05 regarded as statistically significant. Univariate analysis was only included in the multivariate analysis if it was meaningful.

### Mutation analysis and DNA methylation analysis

Mutation analysis was performed in the cBioPortal database (http://www.cbioportal.org). In this study, the genetic alterations and mutations of the ARHGAP family genes in PAAD tissues were analyzed by searching cBioPortal. The cBioPortal database provides analysis of genomic alteration data on more than 200 cancer patients. Select "Pancreas" cancer type, we choose TCGA, Firehose Legacy, and we selected the genomic profiles (mutations, mRNA Expression, and putative copy-number alterations from GISTIC). Finally, we entered each gene name in the query box and obtained the genetic alterations of the ARHGAPs. The DNA methylation of ARHGAP family genes was analyzed using the UALCAN database (https://ualcan.path.uab.edu). UALCAN is a user-friendly and comprehensive portal website, which provides insight into TCGA gene methylation data. We used the TCGA transcription level to analyze, input gene symbol, select pancreatic cancer data, and finally select methylation links for analysis to get the methylation level of the gene.

### Immune infiltration analysis

The Tumor Immune Estimation Resource (TIMER) is a web server for analyzing the abundance of tumor infiltrates, offering immune strategies and targeting molecules clues. Using TIMER (https://cistrome.shinyapps.io/timer/), we investigated the relationship between the expression of ARHGAP family genes and the infiltration levels of B cells, CD4 + T cells, CD8 + T cells, macrophages, neutrophils, and other immune cells. The deconvolution algorithm CIBERSORT, which is a means of computing cell composition based on the expression profiles, was used to calculate the proportion of 22 immune cells in each patient with PAAD, the sum of 22 immune cell type fractions in each sample was 1. Based on the application of the single-sample gene set enrichment analysis (ssGSEA) method from the R package GSVA. According to the gene expression levels in 28 published immune cell gene sets, we calculated the degree of infiltration of 28 immune cell types.

### Functional enrichment analysis

We explored the gene functions and potential signaling pathways in tumorigenesis and progression in PAAD using the R package “clusterprofiler” to carry out Gene Ontology (GO) enrichment analysis and Kyoto Encyclopedia of Genes and Genomes (KEGG) analysis^33–35^. The data come from the ARHGAP family genes and 100 ARHGAP-related genes in the GEPIA2 database. GO analysis was divided into three parts: BP (biological process), CC (cellular component), and MF (molecular function). A P-value less than 0.05 was considered statistically significant.

### Sample collection

Human PAAD tissues and corresponding adjacent tissues were selected as tissue specimens from 65 patients with pancreatic cancer diagnosed by pathology at First People’s Hospital of Lianyungang from 2018 to 2022. Clinical staging of the pancreatic cancers was based on the TNM staging criteria of the 8th edition of the International Union for Cancer Control (UICC). All the selected patients had complete clinical and pathological data, and none had received any tumor treatment before their operations.

### Immunohistochemistry staining

The resected pancreatic cancer and adjacent tissue samples were fixed with 4% paraformaldehyde, embedded in paraffin, and then cut into 4 µm thick sections. The slices were then dehydrated, fixed, and covered with a cover glass. The slices were deparaffinized in xylene and then rehydrated in an ethanol gradient. Endogenous peroxidase activity was blocked with 3% hydrogen peroxide, and 3% goat serum was used for nonspecific binding. The primary antibody was added dropwise to the tissue section at the following dilution ratios: ARHGAP5 (1:200, FNab00553, FineTest), ARHGAP11A (1:200, NBP1-93657, NOVUS), and ARHGAP12 (1:200, 201871-T40, Sino). The samples were incubated overnight at 4 °C and then rewarmed for 30 min. The primary antibody was removed, and the sections were sealed for 10 min and washed with PBS. The sections were then incubated with secondary antibody, and 3,3’-diaminohydrazine (DAB) was added to develop the color. The sections were counterstained with hematoxylin, dehydrated, cleared, sealed with neutral glue, and observed under a microscope^36^. The semi-quantitative integration method is used for scoring, which involves multiplying the proportion of tumor cells with positive expression in the entire microscopic field of view by the staining intensity score. Two pathologists, unaware of the clinical information, select tumor cell areas with uniform staining effects for scoring. They observe and score 5 to 10 stained tissue fields under 100 × and 200 × magnification. Immunoreactivity was scored by the percentage of the stained cells (0, no staining; 1, 0–25%; 2, 25–50%; 3, 50–75%; 4, ≥ 75%) and the intensity of staining in cellular plasma, membrane and nuclear (0, no color; 1, slight yellow; 2, yellow brown; 3, brown). The product of the gene positive cell ratio and staining intensity determines the final score. Scores below 5 are defined as negative low expression, while scores above 5 are defined as positive high expression.

### Immunotherapy outcome prediction

The Cancer Immunome Atlas (https://tcia.at/) was used to characterize the intratumoral immune landscapes and the cancer antigenomes from 20 solid cancers. The immunophenotype score (IPS) data of patients with TCGA-PAAD were extracted and used for subsequent analyses to predict the response to immunotherapy, including treatment with cytotoxic T-lymphocyte antigen-4 (CTLA-4) and programmed cell death protein 1 (PD-1) blockers.

### Targeted drug therapy outcome prediction

We predicted the targeted drug therapy outcomes of PAAD patients based on the public pharmacogenomics database Genomics of Drug Sensitivity in Cancer (GDSC; https://www.cancerrxgene.org). The half maximum inhibitory concentration (IC_50_) estimated by the R package “oncoPredict” was used for erlotinib, gemcitabine, olaparib, paclitaxel, oxaliplatin, irinotecan, cisplatin, and 5-fluorouracil.

### Statistical analysis

Statistical analysis was conducted using SPSS software (SPSS 25.0) and R software (version 4.2.2). The differences between two or more groups were compared using Student’s *t*-test. The correlations between ARHGAP family genes and the clinicopathological characteristics of PAAD patients were analyzed using the chi-square test and Fisher’s exact probability method. During the entire study, the statistical significance threshold was P < 0.05.

### Ethics approval and consent to participate

This study was approved by the Ethical Committee of First People’s Hospital of Lianyungang (KY20190924002), and informed consent was obtained from all subjects and/or their legal guardian(s). All the experiments in your study were conducted in accordance to the relevant guidelines and regulations or in accordance to the Declaration of Helsinki is missing.

### Supplementary Information

Supplementary Table S1.

Supplementary Table S2.

Supplementary Table S3.

Supplementary Table S4.

## Data Availability

The data comes from TCGA (https://xenabrowser.net/datapages/) and GEO (https://www.ncbi.nlm.nih.gov/geo/) public database.
