# Supplementary material for: Integrated analysis identified the role of three family members of ARHGAP in pancreatic adenocarcinoma
Source: Sci Rep. 2024 May 23;14:11790. doi: 10.1038/s41598-024-62577-z (PMC11116390; doi:10.1038/s41598-024-62577-z)
Supplement: Supplementary file 1 — Supplementary Table S1. [file 41598_2024_62577_MOESM1_ESM.docx]

**Table S1. The information of these top 100 genes of Pearson correlation coefficient of ARHGAP5 and ARHGAP11A**

| ARHGAP5 | ARHGAP11A |
| --- | --- |
| \| Gene Symbol \| Gene ID \| PCC \| \| --- \| --- \| --- \| \| CSNK1G3 \| ENSG00000151292.17 \| 0.91 \| \| PPP2R5E \| ENSG00000154001.13 \| 0.9 \| \| MPP5 \| ENSG00000072415.8 \| 0.9 \| \| RBM27 \| ENSG00000091009.7 \| 0.9 \| \| ITCH \| ENSG00000078747.12 \| 0.9 \| \| YY1 \| ENSG00000100811.10 \| 0.9 \| \| G2E3 \| ENSG00000092140.14 \| 0.9 \| \| GMFB \| ENSG00000197045.12 \| 0.9 \| \| RPS6KA3 \| ENSG00000177189.12 \| 0.89 \| \| ZFP91 \| ENSG00000186660.14 \| 0.89 \| \| GTF2A1 \| ENSG00000165417.11 \| 0.89 \| \| PUM1 \| ENSG00000134644.15 \| 0.89 \| \| SPTY2D1 \| ENSG00000179119.14 \| 0.89 \| \| YIPF6 \| ENSG00000181704.11 \| 0.89 \| \| ARHGEF12 \| ENSG00000196914.8 \| 0.89 \| \| ATP7A \| ENSG00000165240.17 \| 0.89 \| \| RP2 \| ENSG00000102218.5 \| 0.89 \| \| AFF4 \| ENSG00000072364.12 \| 0.89 \| \| STRN \| ENSG00000115808.11 \| 0.88 \| \| STRN3 \| ENSG00000196792.11 \| 0.88 \| \| MAGT1 \| ENSG00000102158.19 \| 0.88 \| \| FCF1 \| ENSG00000119616.11 \| 0.88 \| \| RLIM \| ENSG00000131263.12 \| 0.88 \| \| TOX4 \| ENSG00000092203.13 \| 0.88 \| \| SNX6 \| ENSG00000129515.18 \| 0.88 \| \| STAG2 \| ENSG00000101972.18 \| 0.88 \| \| SP3 \| ENSG00000172845.13 \| 0.88 \| \| KLHL28 \| ENSG00000179454.13 \| 0.88 \| \| SRFBP1 \| ENSG00000151304.5 \| 0.88 \| \| RBM12 \| ENSG00000244462.7 \| 0.88 \| \| RAB6A \| ENSG00000175582.19 \| 0.88 \| \| G3BP2 \| ENSG00000138757.14 \| 0.88 \| \| WDR44 \| ENSG00000131725.13 \| 0.88 \| \| SPOPL \| ENSG00000144228.8 \| 0.88 \| \| NAA30 \| ENSG00000139977.13 \| 0.88 \| \| MAP3K2 \| ENSG00000169967.16 \| 0.88 \| \| EIF4E \| ENSG00000151247.12 \| 0.88 \| \| ABI1 \| ENSG00000136754.16 \| 0.88 \| \| RAB10 \| ENSG00000084733.10 \| 0.88 \| \| UEVLD \| ENSG00000151116.16 \| 0.88 \| \| ATAD1 \| ENSG00000138138.13 \| 0.88 \| \| TROVE2 \| ENSG00000116747.12 \| 0.88 \| \| ZBTB41 \| ENSG00000177888.7 \| 0.88 \| \| NUMB \| ENSG00000133961.19 \| 0.88 \| \| PTBP3 \| ENSG00000119314.15 \| 0.88 \| \| G3BP1 \| ENSG00000145907.14 \| 0.88 \| \| WASL \| ENSG00000106299.7 \| 0.87 \| \| ZNF562 \| ENSG00000171466.9 \| 0.87 \| \| CAPZA2 \| ENSG00000198898.12 \| 0.87 \| \| SEC24B \| ENSG00000138802.11 \| 0.87 \| \| TLK1 \| ENSG00000198586.13 \| 0.87 \| \| ZNF146 \| ENSG00000167635.11 \| 0.87 \| \| CASC4 \| ENSG00000166734.18 \| 0.87 \| \| GCC2 \| ENSG00000135968.19 \| 0.87 \| \| YWHAZP4 \| ENSG00000213131.3 \| 0.87 \| \| KRAS \| ENSG00000133703.11 \| 0.87 \| \| RAB5A \| ENSG00000144566.10 \| 0.87 \| \| CNIH1 \| ENSG00000100528.11 \| 0.87 \| \| PGGT1B \| ENSG00000164219.9 \| 0.87 \| \| GPD2 \| ENSG00000115159.15 \| 0.87 \| \| PPP3CA \| ENSG00000138814.16 \| 0.87 \| \| MAPK8 \| ENSG00000107643.15 \| 0.87 \| \| FAM199X \| ENSG00000123575.8 \| 0.87 \| \| CREB1 \| ENSG00000118260.14 \| 0.87 \| \| ATF2 \| ENSG00000115966.16 \| 0.87 \| \| UBE2D3 \| ENSG00000109332.19 \| 0.87 \| \| CNOT6 \| ENSG00000113300.11 \| 0.87 \| \| NF1 \| ENSG00000196712.16 \| 0.87 \| \| ZNF148 \| ENSG00000163848.18 \| 0.87 \| \| EFR3A \| ENSG00000132294.13 \| 0.87 \| \| TMEM184C \| ENSG00000164168.7 \| 0.87 \| \| SLC25A24 \| ENSG00000085491.15 \| 0.87 \| \| PUM2 \| ENSG00000055917.15 \| 0.87 \| \| SMEK2 \| ENSG00000275052.4 \| 0.87 \| \| DNAJB14 \| ENSG00000164031.16 \| 0.87 \| \| NAA15 \| ENSG00000164134.12 \| 0.87 \| \| HIPK1 \| ENSG00000163349.21 \| 0.87 \| \| BTBD7 \| ENSG00000011114.14 \| 0.87 \| \| ATP11B \| ENSG00000058063.15 \| 0.87 \| \| HMGXB4 \| ENSG00000100281.13 \| 0.87 \| \| CSNK1A1 \| ENSG00000113712.16 \| 0.87 \| \| ACBD3 \| ENSG00000182827.8 \| 0.87 \| \| CTTNBP2NL \| ENSG00000143079.14 \| 0.87 \| \| C1D \| ENSG00000197223.11 \| 0.87 \| \| SERINC3 \| ENSG00000132824.13 \| 0.87 \| \| CFAP97 \| ENSG00000164323.12 \| 0.87 \| \| YTHDF3 \| ENSG00000185728.16 \| 0.87 \| \| C16orf72 \| ENSG00000182831.11 \| 0.87 \| \| FAM91A1 \| ENSG00000176853.15 \| 0.87 \| \| PPP2CA \| ENSG00000113575.9 \| 0.87 \| \| CHMP2B \| ENSG00000083937.8 \| 0.87 \| \| HNRNPK \| ENSG00000165119.18 \| 0.87 \| \| RBM7 \| ENSG00000076053.10 \| 0.87 \| \| ROCK2 \| ENSG00000134318.13 \| 0.87 \| \| ASXL2 \| ENSG00000143970.16 \| 0.87 \| \| SEPT10 \| ENSG00000186522.14 \| 0.87 \| \| TMED7 \| ENSG00000134970.13 \| 0.87 \| \| WWP1 \| ENSG00000123124.13 \| 0.87 \| \| TNPO1 \| ENSG00000083312.17 \| 0.87 \| \| USP38 \| ENSG00000170185.9 \| 0.87 \| | \| Gene Symbol \| Gene ID \| PCC \| \| --- \| --- \| --- \| \| NUSAP1 \| ENSG00000137804.12 \| 0.89 \| \| KIF20B \| ENSG00000138182.14 \| 0.89 \| \| ATAD2 \| ENSG00000156802.12 \| 0.87 \| \| BUB1 \| ENSG00000169679.14 \| 0.86 \| \| RACGAP1 \| ENSG00000161800.12 \| 0.85 \| \| LMNB1 \| ENSG00000113368.11 \| 0.85 \| \| KIF11 \| ENSG00000138160.5 \| 0.85 \| \| CENPI \| ENSG00000102384.13 \| 0.85 \| \| ZNF367 \| ENSG00000165244.6 \| 0.84 \| \| CDK1 \| ENSG00000170312.15 \| 0.84 \| \| SMC2 \| ENSG00000136824.18 \| 0.84 \| \| SGOL2 \| ENSG00000163535.17 \| 0.83 \| \| DLGAP5 \| ENSG00000126787.12 \| 0.83 \| \| HMMR \| ENSG00000072571.19 \| 0.83 \| \| CLSPN \| ENSG00000092853.13 \| 0.83 \| \| BUB1B \| ENSG00000156970.12 \| 0.83 \| \| MCM10 \| ENSG00000065328.16 \| 0.83 \| \| INCENP \| ENSG00000149503.12 \| 0.83 \| \| MCM4 \| ENSG00000104738.16 \| 0.82 \| \| CKAP2L \| ENSG00000169607.12 \| 0.82 \| \| STIL \| ENSG00000123473.15 \| 0.82 \| \| CEP55 \| ENSG00000138180.15 \| 0.82 \| \| ZWILCH \| ENSG00000174442.11 \| 0.82 \| \| RAD51 \| ENSG00000051180.16 \| 0.82 \| \| KIF14 \| ENSG00000118193.11 \| 0.82 \| \| KIAA1524 \| ENSG00000163507.13 \| 0.82 \| \| MAD2L1 \| ENSG00000164109.13 \| 0.81 \| \| NUDCD1 \| ENSG00000120526.10 \| 0.81 \| \| SMC4 \| ENSG00000113810.15 \| 0.81 \| \| CCNA2 \| ENSG00000145386.9 \| 0.81 \| \| TMOD3 \| ENSG00000138594.12 \| 0.81 \| \| DEPDC1 \| ENSG00000024526.16 \| 0.81 \| \| ERCC6L \| ENSG00000186871.6 \| 0.81 \| \| NAA50 \| ENSG00000121579.12 \| 0.81 \| \| CENPE \| ENSG00000138778.11 \| 0.81 \| \| OIP5 \| ENSG00000104147.8 \| 0.81 \| \| CCNE2 \| ENSG00000175305.16 \| 0.81 \| \| KIF18A \| ENSG00000121621.6 \| 0.81 \| \| CHEK1 \| ENSG00000149554.12 \| 0.81 \| \| KIF20A \| ENSG00000112984.11 \| 0.8 \| \| PARPBP \| ENSG00000185480.11 \| 0.8 \| \| KIF23 \| ENSG00000137807.13 \| 0.8 \| \| MTBP \| ENSG00000172167.7 \| 0.8 \| \| PRR11 \| ENSG00000068489.12 \| 0.8 \| \| CASC5 \| ENSG00000137812.19 \| 0.8 \| \| GINS4 \| ENSG00000147536.11 \| 0.8 \| \| CENPL \| ENSG00000120334.15 \| 0.8 \| \| NDC1 \| ENSG00000058804.11 \| 0.8 \| \| KIF4A \| ENSG00000090889.11 \| 0.8 \| \| RRM2 \| ENSG00000171848.13 \| 0.8 \| \| GINS1 \| ENSG00000101003.9 \| 0.8 \| \| AURKA \| ENSG00000087586.17 \| 0.8 \| \| FANCI \| ENSG00000140525.17 \| 0.8 \| \| WDR76 \| ENSG00000092470.11 \| 0.79 \| \| KNSTRN \| ENSG00000128944.13 \| 0.79 \| \| TFAM \| ENSG00000108064.10 \| 0.79 \| \| DLAT \| ENSG00000150768.15 \| 0.79 \| \| NCAPG2 \| ENSG00000146918.19 \| 0.79 \| \| SHCBP1 \| ENSG00000171241.8 \| 0.79 \| \| ARHGAP11B \| ENSG00000187951.10 \| 0.79 \| \| MSH2 \| ENSG00000095002.12 \| 0.79 \| \| SUV39H2 \| ENSG00000152455.15 \| 0.79 \| \| CKAP2 \| ENSG00000136108.14 \| 0.79 \| \| ASPM \| ENSG00000066279.16 \| 0.79 \| \| RRM1 \| ENSG00000167325.14 \| 0.79 \| \| CPSF2 \| ENSG00000165934.12 \| 0.79 \| \| ZWINT \| ENSG00000122952.16 \| 0.79 \| \| CDC6 \| ENSG00000094804.9 \| 0.79 \| \| SPDL1 \| ENSG00000040275.16 \| 0.79 \| \| SGOL1 \| ENSG00000129810.14 \| 0.79 \| \| HAUS2 \| ENSG00000137814.9 \| 0.78 \| \| TAF2 \| ENSG00000064313.11 \| 0.78 \| \| NEIL3 \| ENSG00000109674.3 \| 0.78 \| \| G2E3 \| ENSG00000092140.14 \| 0.78 \| \| MTDH \| ENSG00000147649.9 \| 0.78 \| \| DBF4 \| ENSG00000006634.7 \| 0.78 \| \| CTDSPL2 \| ENSG00000137770.13 \| 0.78 \| \| TTK \| ENSG00000112742.9 \| 0.78 \| \| MMS22L \| ENSG00000146263.11 \| 0.78 \| \| YY1 \| ENSG00000100811.10 \| 0.78 \| \| CKAP5 \| ENSG00000175216.14 \| 0.78 \| \| NBN \| ENSG00000104320.13 \| 0.78 \| \| ATP6V1C1 \| ENSG00000155097.11 \| 0.78 \| \| DCUN1D1 \| ENSG00000043093.13 \| 0.78 \| \| DTL \| ENSG00000143476.17 \| 0.78 \| \| NDUFS1 \| ENSG00000023228.13 \| 0.77 \| \| CSTF2 \| ENSG00000101811.13 \| 0.77 \| \| RFC3 \| ENSG00000133119.12 \| 0.77 \| \| DIAPH3 \| ENSG00000139734.17 \| 0.77 \| \| RAD21 \| ENSG00000164754.12 \| 0.77 \| \| FCF1 \| ENSG00000119616.11 \| 0.77 \| \| PCNA \| ENSG00000132646.10 \| 0.77 \| \| PRC1 \| ENSG00000198901.13 \| 0.77 \| \| NEK2 \| ENSG00000117650.12 \| 0.77 \| \| RAD18 \| ENSG00000070950.9 \| 0.77 \| \| CENPN \| ENSG00000166451.13 \| 0.77 \| \| ADAM10 \| ENSG00000137845.14 \| 0.77 \| \| DDIAS \| ENSG00000165490.12 \| 0.77 \| \| PPAT \| ENSG00000128059.8 \| 0.77 \| \| DEK \| ENSG00000124795.14 \| 0.77 \| |

PCC: Pearson Correlation Coefficient
