# Supplementary material for: Integrated analysis identified the role of three family members of ARHGAP in pancreatic adenocarcinoma
Source: Sci Rep. 2024 May 23;14:11790. doi: 10.1038/s41598-024-62577-z (PMC11116390; doi:10.1038/s41598-024-62577-z)
Supplement: Supplementary file 2 — Supplementary Table S2. [file 41598_2024_62577_MOESM2_ESM.docx]

**Table S2.** **Clinical patient information**

| **ID** | **age** | **gender** | **tumor size(cm)** | **lymph node metastasis** | **metastasis** | **tumor stage** | **differentiation degree** | **perineural invasion** | **os(months)** | **state** |
| --- | --- | --- | --- | --- | --- | --- | --- | --- | --- | --- |
| 6900113934 | 61 | male | 3.5 | Yes | No | IIB | Intermediate | No | 12 | Dead |
| 1110341994 | 57 | male | 2.8 | Yes | No | IIB | High | Yes | 13 | Alive |
| 1110263658 | 75 | male | 3.6 | No | Yes | Ⅳ | Low | No | 9 | Dead |
| 1110391503 | 66 | female | 2 | No | No | IA | High | Yes | 17 | Dead |
| 1110252339 | 76 | female | 2.9 | No | Yes | Ⅳ | Low | No | 6 | Dead |
| 4010264285 | 64 | female | 3.3 | No | No | IB | Intermediate | No | 27 | Dead |
| 1110482181 | 62 | female | 4.1 | Yes | No | IIB | High | Yes | 11 | Alive |
| 6900784161 | 64 | male | 3.4 | No | No | IB | Intermediate | No | 10 | Dead |
| 1110411904 | 76 | male | 2.2 | No | No | IB | High | Yes | 18 | Alive |
| 4010389260 | 83 | female | 2.8 | Yes | No | III | High | No | 9 | Dead |
| 4010543717 | 47 | female | 1.8 | No | No | IA | High | Yes | 17 | Dead |
| 1110544580 | 72 | male | 3 | No | No | IB | Intermediate | Yes | 5 | Alive |
| 1110532859 | 71 | male | 3.2 | Yes | No | IIB | Intermediate | Yes | 12 | Alive |
| 4010170638 | 64 | male | 2.6 | No | No | IB | High | Yes | 14 | Alive |
| 4010455681 | 65 | female | 2.2 | No | No | IB | High | No | 16 | Alive |
| 6900032329 | 80 | male | 3 | No | No | IB | Intermediate | No | 9 | Alive |
| 4010122651 | 51 | female | 4.3 | Yes | No | IIB | Intermediate | Yes | 10 | Alive |
| 6900725055 | 75 | female | 4.2 | Yes | No | IIB | Intermediate | Yes | 13 | Alive |
| 1110203802 | 82 | female | 2.6 | No | No | IB | High | No | 17 | Alive |
| 1110445315 | 65 | male | 4.2 | No | No | IIA | High | Yes | 14 | Alive |
| 1110296992 | 65 | male | 3.5 | Yes | No | III | Low | No | 8 | Dead |
| 4010358562 | 43 | female | 4.2 | Yes | No | IIB | Low | Yes | 10 | Dead |
| 1110347486 | 58 | male | 4.1 | No | No | IIA | High | No | 13 | Dead |
| 4010257716 | 82 | female | 4.4 | No | No | IIA | High | Yes | 13 | Dead |
| 1110256522 | 67 | female | 3.7 | Yes | No | III | Intermediate | No | 9 | Dead |
| 1110101092 | 72 | female | 4.3 | No | No | IIA | Low | No | 20 | Alive |
| 1110379831 | 65 | male | 2.9 | Yes | No | III | High | Yes | 21 | Alive |
| 6900489249 | 66 | female | 4.2 | No | No | IIA | High | Yes | 10 | Dead |
| 1110449096 | 70 | male | 3.6 | No | No | IB | Intermediate | Yes | 9 | Dead |
| 1110372196 | 64 | female | 2 | Yes | No | IIB | High | Yes | 22 | Alive |
| 4010310767 | 60 | male | 2.7 | Yes | No | IIB | High | No | 21 | Dead |
| 1110224304 | 66 | male | 1.8 | No | No | IA | Intermediate | No | 24 | Alive |
| 1110287762 | 78 | female | 2.6 | Yes | No | IIB | High | Yes | 17 | Dead |
| 1110338119 | 56 | male | 2.8 | Yes | No | IIB | Intermediate | No | 3 | Dead |
| 1110466333 | 63 | male | 1.7 | No | No | IA | Intermediate | No | 12 | Alive |
| 4010358554 | 59 | male | 2 | No | No | IA | Intermediate | Yes | 7 | Alive |
| 4010754554 | 68 | male | 2.7 | Yes | No | IIB | High | No | 6 | Alive |
| 1110286655 | 48 | female | 4 | No | Yes | Ⅳ | Low | No | 7 | Dead |
| 4010548156 | 51 | male | 4.2 | No | No | IIA | Intermediate | Yes | 11 | Alive |
| 1110499442 | 61 | male | 3.5 | Yes | No | IIB | High | Yes | 15 | Alive |
| 6900109841 | 68 | male | 4.4 | Yes | No | III | Intermediate | Yes | 6 | Alive |
| 1110225272 | 68 | male | 3.3 | Yes | No | IIB | Low | No | 5 | Dead |
| 6900528375 | 69 | male | 2 | No | No | IA | High | No | 20 | Alive |
| 4010434839 | 61 | male | 1.8 | Yes | No | IIB | Low | No | 7 | Alive |
| 4010742958 | 52 | male | 4.2 | No | No | IIA | Intermediate | Yes | 5 | Alive |
| 1110087311 | 66 | female | 1.6 | Yes | No | IIB | Intermediate | No | 15 | Alive |
| 4010550999 | 72 | female | 4.3 | No | No | IIA | Intermediate | No | 13 | Dead |
| 1410032218 | 53 | male | 2 | No | No | IA | High | No | 16 | Alive |
| 6900118950 | 76 | female | 3.3 | No | No | IIA | Low | No | 22 | Alive |
| 1110020415 | 39 | male | 3.7 | Yes | No | IIB | Intermediate | Yes | 11 | Alive |
| 6900237029 | 68 | female | 3.5 | Yes | No | IIB | Intermediate | Yes | 9 | Alive |
| 1110373916 | 66 | female | 2.8 | Yes | No | III | High | Yes | 8 | Dead |
| 1110423964 | 77 | male | 3.1 | No | No | IB | Intermediate | Yes | 12 | Alive |
| 6000806345 | 67 | female | 1.6 | No | No | IA | Intermediate | No | 24 | Dead |
| 6900498249 | 58 | male | 2.4 | No | No | IB | Intermediate | No | 10 | Alive |
| 1110462635 | 64 | male | 2.9 | Yes | No | III | Intermediate | Yes | 6 | Dead |
| 1110275129 | 69 | female | 4.2 | No | No | IIA | Intermediate | No | 11 | Alive |
| 4010272657 | 61 | male | 4 | No | Yes | Ⅳ | Low | Yes | 7 | Alive |
| 6900505257 | 74 | male | 3.4 | Yes | No | IIB | Intermediate | No | 11 | Dead |
| 6900057139 | 74 | male | 2.2 | No | No | IB | High | No | 14 | Alive |
| 1110413154 | 62 | male | 3.8 | No | Yes | Ⅳ | Low | Yes | 9 | Dead |
| 4010644402 | 64 | male | 3.3 | Yes | No | III | High | Yes | 10 | Dead |
| 4010422782 | 57 | female | 3.5 | Yes | No | III | Intermediate | Yes | 12 | Dead |
| 6900017532 | 74 | male | 2.5 | No | No | IB | Intermediate | No | 14 | Alive |
| 6000072444 | 75 | male | 1.8 | No | No | IA | Intermediate | No | 12 | Alive |
