# Supplementary material for: Integrated analysis identified the role of three family members of ARHGAP in pancreatic adenocarcinoma
Source: Sci Rep. 2024 May 23;14:11790. doi: 10.1038/s41598-024-62577-z (PMC11116390; doi:10.1038/s41598-024-62577-z)
Supplement: Supplementary file 3 — Supplementary Table S3. [file 41598_2024_62577_MOESM3_ESM.docx]

**Table S3. The information of the patients in three databases**

| Data set | Year | Platform | Participants | | Organization name | Samples |
| --- | --- | --- | --- | --- | --- | --- |
|  |  |  | Tumor | Normal |  |  |
| GSE28735 | 2012 | GPL6244 | 45 | 45 | NCI/NIH | GSM711904  GSM711905  GSM711906  GSM711907  GSM711908 |
| GSE16515 | 2009 | GPL570 | 36 | 16 | Mayo Clinic | GSM414924  GSM414925  GSM414926  GSM414927  GSM414928 |
| GSE15471 | 2009 | GPL570 | 39 | 39 | ICI | GSM388076  GSM388077  GSM388078  GSM388079  GSM388080 |
| TCGA | 2017 | IlluminaHiSeq_RNASeqV2 | 178 | 4 | University of North Carolina | TCGA-2L-AAQL-01 TCGA-2J-AABI-01 TCGA-3A-A9J0-01 TCGA-3A-A9I7-01 TCGA-2J-AABO-01 |
