# Supplementary material for: Integrated analysis identified the role of three family members of ARHGAP in pancreatic adenocarcinoma
Source: Sci Rep. 2024 May 23;14:11790. doi: 10.1038/s41598-024-62577-z (PMC11116390; doi:10.1038/s41598-024-62577-z)
Supplement: Supplementary file 4 — Supplementary Table S4. [file 41598_2024_62577_MOESM4_ESM.docx]

**Table S4. Correspondence between ARHGAPs gene expression and type of immune infiltration based on immuno-related markers in PAAD**

| **Description** | **Markers** | **ARHGAP5** | | | | **ARHGAP11A** | | | | **ARHGAP12** | | | |
| --- | --- | --- | --- | --- | --- | --- | --- | --- | --- | --- | --- | --- | --- |
|  |  | **None** | | **Purity** | | **None** | | **Purity** | | **None** | | **Purity** | |
|  |  | **Cor** | **P** | **Cor** | **P** | **Cor** | **P** | **Cor** | **P** | **Cor** | **P** | **Cor** | **P** |
| B cell | CD19 | -0.185 | **1.31E-02** | -0.175 | **2.15E-02** | -0.006 | 9.33E-01 | -0.059 | 4.45E-01 | -0.095 | 2.06E-01 | -0.122 | 1.12E-01 |
|  | CD79A | -0.167 | **2.49E-02** | -0.155 | **4.28E-02** | 0.002 | 9.81E-01 | -0.060 | 4.35E-01 | -0.086 | 2.50E-01 | -0.113 | 1.42E-01 |
| T cell(general) | CD2 | -0.013 | 8.60E-01 | -0.006 | 9.30E-01 | -0.030 | 6.87E-01 | -0.082 | 2.86E-01 | -0.018 | 8.16E-01 | -0.041 | 5.97E-01 |
|  | CD3D | -0.097 | 1.95E-01 | -0.098 | 2.02E-01 | -0.042 | 5.73E-01 | -0.093 | 2.26E-01 | -0.070 | 3.54E-01 | -0.095 | 2.16E-01 |
|  | CD3E | -0.047 | 5.24E-01 | -0.044 | 5.59E-01 | -0.041 | 5.90E-01 | -0.091 | 2.39E-01 | -0.055 | 4.65E-01 | -0.081 | 2.92E-01 |
| CD8+ T cell | CD8A | 0.007 | 9.17E-01 | 0.017 | 8.22E-01 | -0.007 | 9.31E-01 | -0.046 | 5.54E-01 | -0.033 | 6.57E-01 | -0.047 | 5.38E-01 |
|  | CD8B | 0.010 | 8.92E-01 | 0.025 | 7.37E-01 | 0.021 | 7.80E-01 | -0.027 | 7.26E-01 | -0.053 | 4.93E-01 | -0.035 | 6.42E-01 |
| Monocyte | CD86 | 0.101 | 1.77E-01 | 0.125 | 1.02E-01 | 0.137 | 6.73E-02 | 0.101 | 1.90E-01 | -0.055 | 4.68E-01 | -0.088 | 2.55E-01 |
|  | CSF1R | 0.090 | 2.29E-01 | 0.111 | 1.48E-01 | 0.042 | 5.72E-01 | 0.016 | 8.31E-01 | -0.089 | 2.36E-01 | -0.124 | 1.05E-01 |
| TAM | CCL2 | -0.117 | 1.18E-01 | -0.107 | 1.60E-01 | -0.133 | 7.66E-02 | -0.165 | **3.08E-02** | -0.156 | **3.67E-02** | -0.158 | **3.95E-02** |
|  | CD68 | 0.288 | **9.59E-05** | 0.342 | **4.54E-06** | 0.200 | **7.45E-03** | 0.168 | **2.85E-02** | 0.203 | **6.55E-03** | 0.176 | **2.12E-02** |
|  | IL10 | -0.017 | 8.21E-01 | -0.005 | 9.45E-01 | 0.047 | 5.33E-01 | 0.009 | 9.06E-01 | -0.036 | 6.34E-01 | -0.046 | 5.53E-01 |
| M1 Macrophage | INOS(NOS2) | 0.120 | 1.09E-01 | 0.147 | 5.46E-02 | 0.248 | **8.34E-04** | 0.210 | **5.80E-03** | 0.049 | 5.13E-01 | 0.026 | 7.34E-01 |
|  | IRF5 | 0.049 | 5.06E-01 | 0.051 | 5.06E-01 | 0.063 | 4.02E-01 | 0.034 | 6.55E-01 | 0.152 | **4.20E-02** | 0.153 | **4.56E-02** |
|  | COX2(PTGS2) | 0.339 | **3.81E-06** | 0.368 | **6.90E-07** | 0.350 | **1.90E-06** | 0.336 | **7.25E-06** | 0.243 | **1.10E-03** | 0.252 | **8.84E-04** |
| M2 Macrophage | CD163 | 0.158 | **3.36E-02** | 0.185 | **1.52E-02** | 0.177 | **1.76E-02** | 0.153 | **4.50E-02** | -0.030 | 6.86E-01 | -0.066 | 3.94E-01 |
|  | VSIG4 | 0.110 | 1.42E-01 | 0.141 | 6.43E-02 | 0.100 | 1.83E-01 | 0.070 | 3.64E-01 | -0.036 | 6.30E-01 | -0.078 | 3.09E-01 |
|  | MS4A4A | 0.083 | 2.65E-01 | 0.107 | 1.64E-01 | 0.096 | 2.00E-01 | 0.060 | 4.38E-01 | -0.049 | 5.12E-01 | -0.088 | 2.54E-01 |
| Neutrophils | CD66b(CEACAM8) | 0.102 | 1.76E-01 | 0.114 | 1.39E-01 | 0.084 | 2.63E-01 | 0.058 | 4.49E-01 | 0.203 | **6.51E-03** | 0.189 | **1.31E-02** |
|  | CD11b(ITGAM) | 0.145 | 5.28E-02 | 0.183 | **1.66E-02** | 0.152 | **4.28E-02** | 0.109 | 1.56E-01 | 0.077 | 3.08E-01 | 0.046 | 5.54E-01 |
|  | CCR7 | -0.093 | 2.14E-01 | -0.083 | 2.81E-01 | -0.067 | 3.71E-01 | -0.111 | 1.47E-01 | -0.115 | 1.24E-01 | -0.129 | 9.36E-02 |
| Natural killer cell | KIR2DL1 | 0.040 | 5.92E-01 | 0.022 | 7.71E-01 | -0.020 | 7.89E-01 | -0.011 | 8.81E-01 | 0.001 | 9.93E-01 | 0.006 | 9.36E-01 |
|  | KIR2DL3 | 0.088 | 2.39E-01 | 0.095 | 2.17E-01 | 0.121 | 1.07E-01 | 0.102 | 1.83E-01 | 0.013 | 8.67E-01 | 0.012 | 8.78E-01 |
|  | KIR2DL4 | 0.233 | **1.71E-03** | 0.254 | **8.18E-04** | 0.280 | **1.51E-04** | 0.273 | **3.00E-04** | 0.096 | 2.01E-01 | 0.105 | 1.73E-01 |
|  | KIR3DL1 | -0.035 | 6.40E-01 | -0.011 | 8.81E-01 | -0.025 | 7.42E-01 | -0.065 | 3.95E-01 | -0.134 | 7.43E-02 | -0.137 | 7.31E-02 |
|  | KIR3DL2 | 0.048 | 5.23E-01 | 0.062 | 4.20E-01 | 0.101 | 1.80E-01 | 0.070 | 3.60E-01 | 0.038 | 6.17E-01 | 0.023 | 7.62E-01 |
|  | KIR3DL3 | 0.126 | 9.19E-02 | 0.134 | 7.97E-02 | 0.199 | **7.58E-03** | 0.196 | **1.03E-02** | 0.112 | 1.37E-01 | 0.119 | 1.21E-01 |
|  | KIR2DS4 | 0.035 | 6.40E-01 | 0.046 | 5.54E-01 | 0.088 | 2.40E-01 | 0.051 | 5.04E-01 | -0.046 | 5.44E-01 | -0.046 | 5.50E-01 |
| Dendritic cell | HLA-DPB1 | -0.027 | 7.18E-01 | -0.020 | 7.94E-01 | -0.018 | 8.07E-01 | -0.063 | 4.16E-01 | -0.101 | 1.78E-01 | -0.131 | 8.70E-02 |
|  | HLA-DQB1 | 0.053 | 4.81E-01 | 0.067 | 3.82E-01 | 0.139 | 6.37E-02 | 0.111 | 1.49E-01 | -0.020 | 7.91E-01 | -0.036 | 6.45E-01 |
|  | HLA-DRA | 0.092 | 2.22E-01 | 0.108 | 1.59E-01 | 0.115 | 1.24E-01 | 0.081 | 2.91E-01 | 0.006 | 9.33E-01 | -0.019 | 8.02E-01 |
|  | HLA-DPA1 | 0.085 | 2.60E-01 | 0.102 | 1.86E-01 | 0.099 | 1.87E-01 | 0.067 | 3.86E-01 | -0.039 | 5.99E-01 | -0.067 | 3.82E-01 |
|  | BDCA-1(CD1C) | 0.012 | 8.69E-01 | 0.017 | 8.28E-01 | -0.088 | 2.42E-01 | -0.119 | 1.22E-01 | 0.013 | 8.58E-01 | 0.001 | 9.92E-01 |
|  | BDCA-4(NRP1) | 0.426 | **3.92E-09** | 0.454 | **4.37E-10** | 0.195 | **9.13E-03** | 0.207 | **6.67E-03** | 0.102 | 1.73E-01 | 0.085 | 2.67E-01 |
|  | CD11c(ITGAX) | 0.001 | 9.88E-01 | 0.019 | 8.06E-01 | 0.062 | 4.10E-01 | 0.009 | 9.06E-01 | -0.046 | 5.37E-01 | -0.079 | 3.01E-01 |
| Th1 | T-bet(TBX21) | -0.056 | 4.55E-01 | -0.059 | 4.40E-01 | -0.099 | 1.89E-01 | -0.123 | 1.10E-01 | -0.110 | 1.42E-01 | -0.126 | 9.94E-02 |
|  | STAT4 | -0.018 | 8.11E-01 | -0.029 | 7.04E-01 | -0.164 | **2.80E-02** | -0.154 | **4.43E-02** | -0.227 | **2.32E-03** | -0.222 | **3.54E-03** |
|  | STAT1 | 0.386 | **1.24E-07** | 0.419 | **1.14E-08** | 0.456 | **2.11E-10** | 0.447 | **8.89E-10** | 0.360 | **9.15E-07** | 0.359 | **1.44E-06** |
|  | IFNG | 0.026 | 7.30E-01 | 0.043 | 5.73E-01 | 0.163 | **2.93E-02** | 0.144 | 5.99E-02 | -0.019 | 8.03E-01 | -0.025 | 7.49E-01 |
|  | TNF | -0.064 | 3.96E-01 | -0.036 | 6.39E-01 | 0.078 | 2.97E-01 | 0.047 | 5.42E-01 | 0.018 | 8.16E-01 | 0.016 | 8.35E-01 |
| Th2 | GATA3 | 0.199 | **7.53E-03** | 0.217 | **4.29E-03** | 0.188 | **1.18E-02** | 0.165 | **3.08E-02** | 0.215 | **3.99E-03** | 0.209 | **6.03E-03** |
|  | STAT6 | 0.425 | **4.28E-09** | 0.450 | **6.65E-10** | 0.262 | **4.14E-04** | 0.237 | **1.84E-03** | 0.568 | **1.09E-16** | 0.574 | **2.38E-16** |
|  | STAT5A | 0.096 | 2.01E-01 | 0.110 | 1.52E-01 | 0.061 | 4.15E-01 | 0.019 | 8.01E-01 | 0.087 | 2.48E-01 | 0.069 | 3.71E-01 |
|  | IL13 | -0.137 | 6.71E-02 | -0.120 | 1.18E-01 | -0.064 | 3.94E-01 | -0.072 | 3.47E-01 | -0.177 | **1.75E-02** | -0.165 | **3.15E-02** |
| Tfh | BCL6 | 0.396 | **5.32E-08** | 0.408 | **2.94E-08** | 0.233 | **1.72E-03** | 0.219 | **3.94E-03** | 0.303 | **4.04E-05** | 0.307 | **4.52E-05** |
|  | IL21 | -0.040 | 5.96E-01 | -0.028 | 7.14E-01 | 0.017 | 8.26E-01 | -0.019 | 8.02E-01 | -0.096 | 2.00E-01 | -0.103 | 1.78E-01 |
| Th17 | IL17A | 0.073 | 3.31E-01 | 0.074 | 3.39E-01 | 0.043 | 5.64E-01 | 0.033 | 6.65E-01 | 0.098 | 1.90E-01 | 0.089 | 2.48E-01 |
|  | STAT3 | 0.482 | **7.10E-12** | 0.501 | **2.96E-12** | 0.227 | **2.30E-03** | 0.209 | **5.99E-03** | 0.225 | **2.47E-03** | 0.224 | **3.24E-03** |
| Treg | FOXP3 | 0.023 | 7.61E-01 | 0.039 | 6.17E-01 | 0.083 | 2.72E-01 | 0.045 | 5.61E-01 | -0.035 | 6.43E-01 | -0.058 | 4.51E-01 |
|  | CCR8 | 0.182 | **1.50E-02** | 0.206 | **6.79E-03** | 0.200 | **7.35E-03** | 0.164 | **3.20E-02** | 0.094 | 2.11E-01 | 0.078 | 3.12E-01 |
|  | STAT5B | 0.358 | **1.01E-06** | 0.367 | **8.16E-07** | 0.010 | 8.98E-01 | 0.038 | 6.18E-01 | 0.055 | 4.62E-01 | 0.089 | 2.49E-01 |
|  | TGFB1 | 0.022 | 7.65E-01 | 0.024 | 7.52E-01 | 0.081 | 2.84E-01 | 0.050 | 5.13E-01 | -0.130 | 8.25E-02 | -0.147 | 5.49E-02 |
| T cell exhaustion | TIM3(HAVCR2) | 0.086 | 2.53E-01 | 0.111 | 1.48E-01 | 0.105 | 1.63E-01 | 0.068 | 3.74E-01 | -0.027 | 7.24E-01 | -0.061 | 4.29E-01 |
|  | GZMB | -0.036 | 6.33E-01 | -0.023 | 7.68E-01 | 0.143 | 5.62E-02 | 0.091 | 2.36E-01 | -0.093 | 2.15E-01 | -0.125 | 1.04E-01 |
|  | LAG3 | -0.055 | 4.63E-01 | -0.054 | 4.85E-01 | 0.087 | 2.49E-01 | 0.078 | 3.11E-01 | -0.154 | **4.00E-02** | -0.150 | **4.98E-02** |
